# The revised-risk analysis index as a predictor of major morbidity and mortality in older patients after abdominal surgery: a retrospective cohort study

**DOI:** 10.1186/s12871-022-01844-w

**Published:** 2022-09-22

**Authors:** Bin Wei, Yanan Zong, Mao Xu, Xiaoxiao Wang, Xiangyang Guo

**Affiliations:** 1 Department of Anesthesiology, Peking University Third Hospital, 49 North Gardon Rd., Haidian District, Beijing, 100191 P.R. China; 2 Clinical Epidemiology Research Center, Peking University Third Hospital, Beijing, China

**Keywords:** Frailty, Revised-risk analysis index, Morbidity, Mortality, Older patient, Surgery

## Abstract

**Background:**

The revised-Risk Analysis Index (RAI-rev) can accurately predict postoperative mortality risk. However, the association of RAI-rev with composite outcome of major morbidity and mortality (MMM) among older surgical patients is largely unknown. This study investigated the association between RAI-rev and postoperative MMM in older patients undergoing abdominal surgery. It also assessed the predictive value of RAI-rev combined with other preoperative risk factors.

**Methods:**

This retrospective cohort study reviewed the medical records of all patients aged 65 and older who underwent abdominal surgery between January 2018 and December 2019. The primary outcome was the postoperative MMM during hospitalization, and its association with preoperative RAI-rev scores was assessed using multivariable logistic regression analysis. The prediction of postoperative outcomes was used the receiver-operating characteristic curve analysis.

**Results:**

A total of 2225 older patients were analyzed, and 258 (11.6%) developed postoperative MMM. After adjusting for confounders, each unit increase in RAI-rev scores resulted in a 2.3% increase in the MMM risk and a 3.0% increase in the odds of life-threatening complications and mortality (both *P* < 0.05). The area under the curves (AUCs) of RAI-rev scores in predicting MMM and life-threatening complications and mortality was 0.604 (95% CI: 0.567 to 0.640) and 0.633 (95% CI: 0.592 to 0.675), respectively (both *P* < 0.001); when the RAI-rev was combined with age, gender, American Society of Anesthesiologists (ASA) classification, operative stress, and urgency status of surgery (emergency or elective), the AUCs were 0.694 (95% CI: 0.659 to 0.729) and 0.739 (95% CI: 0.702 to 0.777), respectively (both *P* < 0.001).

**Conclusions:**

Higher RAI-rev scores were independently associated with increased risk of MMM. When combined with age, gender, ASA classification, operative stress, and urgency status of surgery, RAI-rev had improved performance in predicting the risk of MMM, particularly the life-threatening complications and mortality.

**Supplementary Information:**

The online version contains supplementary material available at 10.1186/s12871-022-01844-w.

## Background

With a significant increase in life expectancy, the proportion of older adults (≥65 years) has increased dramatically over the past decades [1]. This has led to an increasing number of older people undergoing surgical procedures, which presents a huge challenge for clinicians. Despite improvement in perioperative medical care and management, older patients still have higher odds of postoperative complications and mortality than younger patients [2, 3]. Therefore, it is imperative for clinicians to accurately stratify the perioperative risks among older patients and provide tailored clinical care to improve postoperative outcomes. Frailty, as a geriatric syndrome characterized by a combination of reduced physiologic reserve and multisystem deficit accumulation distinct from normal aging processes [4], has emerged as a key predictor for adverse outcomes in older surgical patients [2, 3, 5, 6].

Various screening tools have been proposed to measure frailty in perioperative settings, such as the Risk Analysis Index (RAI) developed by Hall and colleagues [7]. The RAI is an instrument based on the deficit accumulation model of frailty and comprises multiple frailty domains, including comorbidities, cognitive ability, social, nutrition, and functional status [7–9]. It represents a more comprehensive measure than other similar frailty instruments (e.g., modified frailty index [mFI]) [9, 10]. Initially, the validation of RAI was limited to the veteran surgical cohorts, with women in the minority [7, 11, 12]; several authors then validated it with external cohorts [9, 13, 14]. Among them, Arya et al. modified the original RAI and proposed the revised-Risk Analysis Index (RAI-rev); furthermore, they found that the RAI-rev had improved discrimination and calibration for mortality over the original RAI in perioperative settings [9]. So far, there has been limited data on the association of RAI-rev with postoperative complications and the predictive value of RAI-rev in predicting the composite outcome of major morbidity and mortality (MMM) in older surgical patients.

The current study aimed to determine the association between the RAI-rev scores and the risk of postoperative MMM in older patients undergoing abdominal surgery. Additionally, we sought to explore the predictive value of RAI-rev, as well as that of the combination of RAI-rev with other baseline risk factors (age, gender, American Society of Anesthesiologists [ASA] physical status classification, type of surgery categorized by operative stress, and urgency status of surgery [emergency or elective]), in predicting the occurrence of MMM. We hypothesized that a higher RAI-rev score was associated with an increased odds of MMM and could accurately predict the occurrence of MMM when combined with other preoperative risk factors.

## Methods

The study protocol was approved by the Biomedical Research Ethics Committee of Peking University Third Hospital, Beijing, China (2022 [158–02]). Due to the retrospective design and that no patient follow-up was performed, the Biomedical Research Ethics Committee of Peking University Third Hospital agreed to waive the written informed consent from the patients. The investigators who performed the data collection were blinded to the objective of the study and received strict training sessions.

### Patient selection

This retrospective cohort study reviewed the electronic medical records of older patients (≥65 years of age) who underwent abdominal surgery (including urologic and general surgical procedures) from January 2018 to December 2019, in Peking University Third Hospital. Patients with incomplete or missing perioperative data were excluded. All personal information on patients was kept confidential.

### Measurement of RAI-rev score

RAI-rev score was calculated by evaluating 11 variables derived from the Veterans Affairs or American College of Surgeons National Surgical Quality Improvement Projects (VASQIP/ACS-NSQIP) datasets, i.e., age, sex, cancer, poor appetite, unintentional weight loss, renal failure, congestive heart failure, shortness of breath, residence other than independent living, cognitive decline, and functional status [7–9]. Total score ranges from 0 to 81, with higher scores indicating more severe frailty. Details on the weight of each item are listed in Supplemental Digital Content (SDC) 1. If a patient experienced more than one surgical procedure during the hospital stay and had multiple preoperative RAI-rev scores, only the first round of the surgery and the corresponding preoperative RAI-rev score were analyzed.

### Covariates

Baseline characteristics not covered by the RAI-rev were gathered, including body mass index (BMI), smoking and drinking status, major comorbidities, ASA physical status classification, and main laboratory test results. Intraoperative factors were also extracted, including type of surgery categorized by operative stress [15], urgency status of surgery (emergency or elective), anesthetic methods, duration of surgery, estimated blood loss, and intraoperative blood transfusion. The operative stress levels of surgical procedures were stratified using the Operative Stress Score (OSS), i.e., OSS1, very low stress; OSS 2, low stress; OSS 3, moderate stress; OSS 4, high stress; and OSS 5, very high stress [15].

### Postoperative outcomes

The primary outcome was the occurrence of MMM during hospitalization, i.e., grade III or greater complications according to the Clavien-Dindo (CD) scoring system (SDC 2) [16]. For patients with multiple complications, we included the most severe complication for analysis. The diagnostic criteria for major complications are listed in SDC 3. The secondary outcome was the development of life-threatening complications and mortality, i.e., CD IV or greater complications.

### Statistical analysis

The baseline and perioperative variables were compared between patients with MMM and those without. Continuous variables were analyzed with the independent samples t-test or Mann-Whitney U test; the Kolmogorov-Smirnov test was performed to check for normality. Categorical variables were analyzed using χ^2^ tests, continuity-corrected χ^2^ tests, or Fisher’s exact tests. Time-to-event outcomes in four different RAI-rev subgroups (stratified by RAI-rev scores: 20–29, 30–39, 40–49, and ≥ 50) were compared by using Kaplan–Meier curves (Log-Rank test). The hazard ratios were estimated with univariate Cox proportional hazard regression models.

Perioperative variables that might be associated with the development of MMM were screened using univariate logistic regression analyses and tested for multicollinearity. Independent variables with *P* values < 0.10 in univariate logistic regression analyses and those considered clinically significant were entered into a multivariable logistic regression model to identify the adjusted association of RAI-rev scores with the MMM risk. Similarly, another multivariable logistic regression model was constructed to investigate the adjusted relationship between RAI-rev scores and life-threatening complications and mortality. The 11 variables included in the RAI-rev were not separately enrolled in either univariate or multivariable analyses. The Hosmer-Lemeshow test was used to confirm the goodness of fit of the multivariable logistic regression models.

The predictive performances of RAI-rev scores alone and the combination of age, gender, RAI-rev scores, ASA classification, operative stress, and urgency status of surgery were assessed using the receiver-operating characteristic (ROC) curve analysis. The area under the curve (AUC) was used to test the discriminative power (ability to classify correctly) of these risk factors for outcomes. Differences between the AUCs were compared using the DeLong’ test. The relevant predictive parameters, including sensitivity, specificity, and positive and negative predictive values (PPV and NPV), were calculated for different thresholds of RAI-rev scores. For all analyses, two-tailed *P* values < 0.05 were considered significantly statistical. All statistical analyses were performed with the SPSS version 26.0 (IBM Corp., Armonk, NY, USA) and MedCalc version 19.05 (Ostend, Belgium).

Although the sample size was not estimated in advance, 258 cases of MMM and 16 independent variables included in the corresponding multivariable logistic regression model, as well as 178 cases of life-threatening complications and mortality and 15 independent variables included in the corresponding multivariable model, meet the requirement of the “ten events per variable” rule [17]. Therefore, the sample size (2225) of our study was sufficient and could guarantee the reliability and validity of the regression estimates.

## Results

### Patient characteristics

**Fig. 1:**
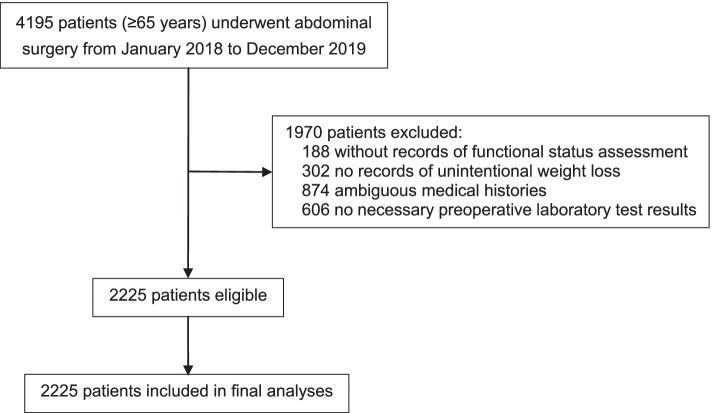
Flowchart of the study

From January 2018 to December 2019, 4195 patients who were ≥ 65 years of age and experienced abdominal surgery were screened. Of these, 1970 patients with missing data on RAI-rev components or other baselined factors (no assessment of preoperative functional status or unintentional weight loss, ambiguous medical histories, or no necessary preoperative laboratory test results) were excluded, leaving 2225 patients for analysis (Fig. 1).

**Fig. 2:**
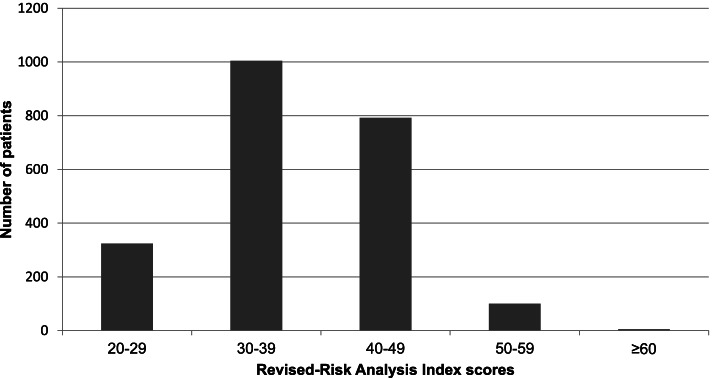
Distribution of the revised-Risk Analysis Index scores in the study patients

The study cohort had a mean age of 73.9 years; 61.4% (1366/2225) were men. The median RAI-rev value of our patients was 38 [IQR: 34 to 42], with most patients having RAI-rev scores between 30 and 39 (Table 1 and Fig. 2). Two hundred fifty-eight patients (11.6%) developed postoperative MMM during hospitalization, of whom 80 (3.6%), 152 (6.8%), and 26 (1.2%) experienced CD grade III, IV complications, and death, respectively (detailed in SDC 3). The median [IQR] RAI-rev score in the patients with MMM was significantly higher than that in those without MMM (41 [37 to 45] vs. 38 [34 to 42], *P* < 0.001). Other baselines and perioperative data are presented in Table 1 and SDC 4.

**Table 1:** Baseline and perioperative characteristics

	All patients (*n* = 2225)	Without MMM (*n* = 1967)	With MMM (*n* = 258)	*P* value
**Demographics**				
Age (years)	73.9 ± 6.4	73.7 ± 6.4	75.3 ± 6.3	**< 0.001**
Body mass index				**< 0.001**
< 18.5 kg/m^2^	162 (7.3%)	124 (6.3%)	38 (14.7%)	
18.5–23.9 kg/m^2^	1141 (51.3%)	1033 (52.5%)	108 (41.9%)	
24–27.9 kg/m^2^	724 (32.5%)	642 (32.6%)	82 (31.8%)	
≥ 28 kg/m^2^	198 (8.9%)	168 (8.5%)	30 (11.6%)	
**Revised-Risk Analysis Index score**	38 [34 to 42]	38 [34 to 42]	41 [37 to 45]	**< 0.001**
Male sex	1366 (61.4%)	1201 (61.1%)	165 (64.0%)	0.369
Age				**0.005**
65–69	689 (31.0%)	635 (32.3%)	54 (20.9%)	
70–74	549 (24.7%)	483 (24.6%)	66 (25.6%)	
75–79	533 (24.0%)	464 (23.6%)	69 (26.7%)	
80–84	310 (13.9%)	261 (13.3%)	49 (19.0%)	
85–89	121 (5.4%)	104 (5.3%)	17 (6.6%)	
> 90	23 (1.0%)	20 (1.0%)	3 (1.2%)	
Cancer	1632 (73.3%)	1449 (73.7%)	183 (70.9%)	0.350
Weight loss ^a^	410 (18.4%)	360 (18.3%)	50 (19.4%)	0.675
Poor appetite	623 (28.0%)	512 (26.0%)	111 (43.0%)	**< 0.001**
Renal failure	23 (1.0%)	16 (0.8%)	7 (2.7%)	**0.012**
Congestive heart failure	27 (1.2%)	17 (0.9%)	10 (3.9%)	**< 0.001**
Short of breath	21 (0.9%)	12 (0.6%)	9 (3.5%)	**< 0.001**
Residence other than independent living	20 (0.9%)	12 (0.6%)	8 (3.1%)	**< 0.001**
Cognitive decline	36 (1.6%)	28 (1.4%)	8 (3.1%)	0.081
Alzheimer’s disease	13 (0.6%)	10 (0.5%)	3 (1.2%)	0.388
Vascular dementia	16 (0.7%)	11 (0.6%)	5 (1.9%)	**0.038**
Parkinson’s disease	9 (0.4%)	7 (0.4%)	2 (0.8%)	0.634
Functional status				**< 0.001**
Totally dependent	77 (3.5%)	45 (2.3%)	32 (12.4%)	
Partially dependent	645 (29.0%)	571 (29.0%)	74 (28.7%)	
Independent	1503 (67.6%)	1351 (68.7%)	152 (58.9%)	
**Preoperative health and comorbidities** ^**b**^				
ASA classification				**< 0.001**
I	15 (0.7%)	13 (0.7%)	2 (0.8%)	
II	1219 (54.8%)	1133 (57.6%)	86 (33.3%)	
III	890 (40.4%)	765 (38.9%)	125 (48.4%)	
IV	101 (4.5%)	56 (2.8%)	45 (17.4%)	
Current smoker/quit ≤7 days	276 (12.4%)	237 (12.0%)	39 (15.1%)	0.160
Current alcoholism	101 (4.5%)	88 (4.5%)	13 (5.0%)	0.682
Hypertension	1122 (50.4%)	983 (50.0%)	139 (53.9%)	0.239
Coronary heart disease	403 (18.1%)	339 (17.2%)	64 (24.8%)	**0.003**
Arrhythmia ^c^	187 (8.4%)	153 (7.8%)	34 (13.2%)	**0.003**
Peripheral vascular disease	236 (10.6%)	200 (10.2%)	36 (14.0%)	0.063
Diabetes mellitus	554 (24.9%)	475 (24.1%)	79 (30.6%)	**0.024**
Chronic obstructive pulmonary disease	148 (6.7%)	124 (6.3%)	24 (9.3%)	0.069
Asthma	48 (2.2%)	44 (2.2%)	4 (1.6%)	0.475
Obstructive sleep apnea ^d^	85 (3.8%)	70 (3.6%)	15 (5.8%)	0.076
Previous stroke	375 (16.9%)	324 (16.5%)	51 (19.8%)	0.184
Stroke with deficits ^e^	92 (4.1%)	75 (3.8%)	17 (6.6%)	**0.035**
Mental disorders ^f^	48 (2.2%)	41 (2.1%)	7 (2.7%)	0.513
Visual/hearing impairment	86 (3.9%)	73 (3.7%)	13 (5.0%)	0.298
Chronic hepatic dysfunction ^g^	113 (5.1%)	89 (4.5%)	24 (9.3%)	**0.001**
Connective tissue disease	37 (1.7%)	33 (1.7%)	4 (1.6%)	> 0.999
Chronic corticosteroid therapy ^h^	77 (3.5%)	64 (3.3%)	13 (5.0%)	0.140
Hyper−/hypothyroidism	43 (1.9%)	35 (1.8%)	8 (3.1%)	0.227
Preoperative infection	141 (6.3%)	104 (5.3%)	37 (14.3%)	**< 0.001**
Anemia ^i^	670 (30.1%)	565 (28.7%)	105 (40.7%)	**< 0.001**
Blood coagulation disorder	44 (2.0%)	38 (1.9%)	6 (2.3%)	0.669
History of DVT or PE	15 (0.7%)	13 (0.7%)	2 (0.8%)	> 0.999
Dyslipidemia	1136 (51.1%)	993 (50.5%)	143 (55.4%)	0.135
Hypoalbuminemia,				**< 0.001**
None	1215 (54.6%)	1104 (56.1%)	111 (43.0%)	
30.0–39.9 g/l	902 (40.5%)	781 (39.7%)	121 (46.9%)	
< 30.0 g/l	108 (4.9%)	82 (4.2%)	26 (10.1%)	
Na^+^ < 135.0 mmol/l	228 (10.2%)	184 (9.4%)	44 (17.1%)	**< 0.001**
**Intra-operative factors**				
Surgery type by Operative Stress Score^j^				**< 0.001**
Very low stress	0 (0.0%)	0 (0.0%)	0 (0.0%)	
Low stress	157 (7.1%)	153 (7.8%)	4 (1.6%)	
Moderate stress	936 (42.1%)	845 (43.0%)	91 (35.3%)	
High stress	1065 (47.9%)	921 (46.8%)	144 (55.8%)	
Very high stress	67 (3.0%)	48 (2.4%)	19 (7.4%)	
Duration of surgery (min)	184 [134 to 246]	179 [133 to 242]	198 [153 to 287]	**< 0.001**
Type of anaesthesia				0.119
General	1225 (55.1%)	1066 (54.2%)	159 (61.6%)	
Combined PNB-general	920 (41.3%)	830 (42.2%)	90 (34.9%)	
Combined epidural-general	69 (3.1%)	62 (3.2%)	7 (2.7%)	
Epidural/combined spinal-epidural	11 (0.5%)	9 (0.5%)	2 (0.8%)	
Emergency surgery	153 (6.9%)	121 (6.2%)	32 (12.4%)	**< 0.001**
Estimated blood loss (ml)	60 [50 to 200]	50 [40 to 150]	100 [50 to 300]	**< 0.001**
Blood transfusion	149 (6.7%)	115 (5.8%)	34 (13.2%)	**< 0.001**
**Postoperative outcomes**				
CD grade III	80 (3.6%)	**–**	80 (3.6%)	**–**
CD grade IV	152 (6.8%)	**–**	152 (58.9%)	**–**
CD grade V	26 (1.2%)	**–**	26 (10.1%)	**–**
ICU admission	643 (28.9%)	445 (22.6%)	198 (76.7%)	**< 0.001**
LOS in ICU (hour)^k^, median (95% CI)	24.0 [21.9 to 26.1]	20.0 [19.3 to 20.7]	96.0 [77.9 to 114.1]	**< 0.001**
Prolonged hospital stay^l^	609 (27.4%)	414 (21.0%)	195 (75.6%)	**< 0.001**
Adverse discharge destination^m^	64 (2.9%)	1 (0.1%)	63 (24.4%)	**< 0.001**

*ASA* American Society of Anesthesiologists, *DVT* Deep venous thrombosis, *PE* Pulmonary embolism, *Na*^*+*^ serum natremia concentration, *PNB* Peripheral nerve block, *CD* Clavien**-**Dindo classification, *ICU* Intensive care unit, *LOS* Length of stay

Data are *n* (%), mean ± SD, or median [IQR]. *P* values in bold indicate < 0.05

^a^ Unintentional weight loss ≥10% from baseline within 6 months, or ≥ 5% within 3 months, or ≥ 2% within 1 month

^b^ Refer to comorbidities that not included in the RAI-rev.

^c^ Arrhythmia that required medical or interventional therapy

^d^ Diagnosed by previous polysomnography, or history inquiry and physical examination, and/or STOP-Bang/Berlin questionnaire

^e^ Excludes vascular dementia

^f^ Include diagnosed depression, anxiety, schizophrenia, phobia, and hallucination

^g^ Refers to hepatic impairment classified as Child-Pugh class B and C

^h^ With a duration of > 1 month

^i^ Diagnosed according to the haemoglobin values from the last laboratory test before surgery, male: < 120 g l^− 1^, female: < 110 g l^− 1^

^j^ Stratified into five categories of physiologic stress, i.e., very low stress, low stress, moderate stress, high stress, and very high stress [15]. Detailed classification of surgery type by Operative Stress Score is provided in Supplemental Digital Content 4

^k^ Analyzed with Kaplan-Meier survival analysis (Log-Rank test)

^l^ Defined as greater than 75th percentiles of LOS in hospital for each type of surgery

^m^ Defined as discharge to destinations other than home (e.g., a long- or short-term care facility)

### Association between RAI-rev scores and MMM

**Fig. 3:**
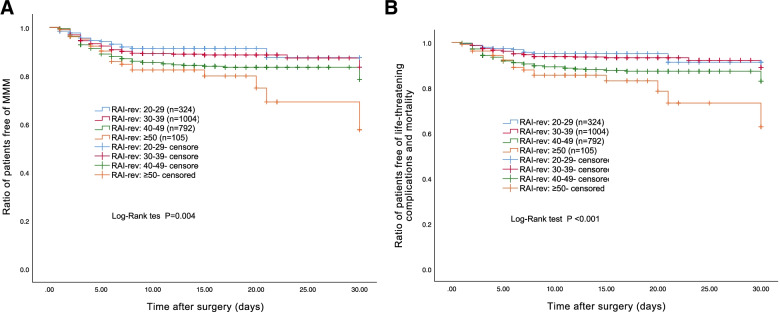
The occurrence of MMM (**A**) and life-threatening complications and mortality (**B**) between four different RAI-rev subgroups (stratified by RAI-rev scores: 20–29, 30–39, 40–49, and ≥ 50). Abbreviations: *MMM* Major morbidity and mortality

There was a significant difference in the occurrence of MMM between the four RAI-rev subgroups (Log-Rank test: *P =* 0.004; Fig. 3A). In the univariate Cox proportional hazard regression analysis, higher RAI-rev scores were associated with a higher rate of MMM (HR: 1.343 per 10-unit increase in scores, 95% CI: 1.141 to 1.580, *P* < 0.001).

The univariate logistic regression analysis also showed that a higher RAI-rev score was associated with an increased risk of MMM, i.e., with every unit increase in the RAI-rev value, the odds of MMM increased by 5.3% (unadjusted OR: 1.053; 95% CI: 1.034 to 1.072; *P* < 0.001). After testing the multicollinearity, 15 other potential risk factors for MMM (*P* < 0.10) were identified by univariate logistic regression analyses (SDC 5 and Table 2). After correcting for the above confounding factors, the rising RAI-rev score remained an independent predictor for an increased risk of MMM, i.e., each unit increase in RAI-rev scores resulted in a 2.3% increase in the odds of MMM (adjusted OR: 1.023; 95% CI: 1.003 to 1.044; *P* = 0.026; Table 2).

**Table 2:** Predictors of postoperative MMM

Variables	Univariable analyses		Multivariable analysis ^a^	
	OR (95% CI)	*P* value	OR (95% CI)	*P* value
Body mass index				
18.5–23.9 kg/m^2^	Reference		Reference	
< 18.5 kg/m^2^	2.931 (1.938 to 4.434)	< 0.001	2.721 (1.758 to 4.212)	< 0.001
≥ 24 kg/m^2^	1.323 (1.000 to 1.750)	0.050	1.332 (0.995 to 1.784)	0.054
Revised-Risk Analysis Index scores	1.053 (1.034 to 1.072)	< 0.001	1.023 (1.003 to 1.044)	0.026
ASA classification				
I/II	Reference		Reference	
III	1.905 (1.435 to 2.528)	< 0.001	1.647 (1.225 to 2.216)	0.001
IV	6.464 (4.191 to 9.971)	< 0.001	5.420 (3.384 to 8.683)	< 0.001
Coronary heart disease	1.584 (1.167 to 2.151)	0.003	–	–
Arrhythmia ^b^	1.800 (1.210 to 2.676)	0.004	–	–
Peripheral vascular disease	1.433 (0.978 to 2.098)	0.065	–	–
Diabetes mellitus	1.386 (1.043 to 1.842)	0.024	–	–
Obstructive sleep apnea ^c^	1.673 (0.943 to 2.968)	0.079	–	–
Stroke with deficits ^d^	1.779 (1.034 to 3.064)	0.038	–	–
Chronic hepatic dysfunction ^e^	2.164 (1.352 to 3.466)	0.001	–	–
Preoperative infection ^f^	2.999 (2.010 to 4.475)	< 0.001	–	–
Anemia ^g^	1.703 (1.304 to 2.224)	< 0.001	–	–
Hypoalbuminemia ^h^				
None	Reference			
30.0–39.9 g/l	1.541 (1.172 to 2.025)	0.002	–	–
< 30.0 g/l	3.154 (1.947 to 5.109)	< 0.001	–	–
Na^+^ < 135.0 mmol/l	1.992 (1.393 to 2.851)	< 0.001	–	–
Surgery type by Operative Stress Score ^i^				
Low stress	Reference		Reference	
Moderate stress	4.119 (1.491 to 11.378)	0.006	2.874 (1.010 to 8.176)	0.048
High stress	5.980 (2.182 to 16.389)	0.001	5.495 (1.940 to 15.570)	0.001
Very high stress	15.141 (4.911 to 46.679)	< 0.001	11.115 (3.419 to 36.138)	< 0.001
Duration of surgery (hour)	1.214 (1.123 to 1.312)	< 0.001	–	–
Emergency surgery	2.160 (1.429 to 3.266)	< 0.001	2.619 (1.603 to 4.278)	< 0.001
Estimated blood loss (100 ml) ^j^	1.052 (1.019 to 1.086)	0.002	–	–
Intra-operative blood transfusion	2.444 (1.627 to 3.672)	< 0.001	1.611 (1.036 to 2.507)	0.034

*ASA* American Society of Anesthesiologists, *Na*^*+*^ serum natremia concentration

^a^ Factors with *P* values < 0.10 in univariate analyses or considered clinically important were included in the multivariable logistic regression model. Age, sex, cancer, poor appetite, unintentional weight loss, renal failure, congestive heart failure, shortness of breath, living status, presence of cognitive decline, and functional status were excluded because they were included in the revised-Risk Analysis Index. The multivariable logistic regression analysis was performed with the backward stepwise method. Hosmer-Lemeshow test for goodness of fit of the multivariable model: *χ*^2^ = 10.908, *df* = 8, *P =* 0.207

^b^ Arrhythmia that required medical or interventional therapy

^c^ Diagnosed by previous polysomnography, or history inquiry and physical examination, and/or STOP-Bang/Berlin questionnaire

^d^ Excludes vascular dementia

^e^ Refers to hepatic impairment classified as Child-Pugh class B and C

^f^ Not included in the multivariable logistic regression analysis because of correlation with emergency surgery

^g^ Diagnosed according to the haemoglobin values from the last laboratory test before surgery, male: < 120 g/l, female: < 110 g/l.

^h^ Not included in the multivariable logistic regression analysis because of correlation with poor appetite

^i^ Stratified into five categories of physiologic stress, i.e., very low stress, low stress, moderate stress, high stress, and very high stress [15]. Detailed classification of surgery type by Operative Stress Score is provided in Supplemental Digital Content 4

^j^ Not included in the multivariable logistic regression analysis because of correlation with intra-operative blood transfusion

### Association between RAI-rev scores and life-threatening complications and mortality

A significant difference was noted in the rates of life-threatening complications and mortality between the four RAI-rev subgroups (Log-Rank test: *P* < 0.001; Fig. 3B). Based on the univariate Cox proportional hazard regression analysis result, higher RAI-rev scores were correlated with a higher rate of life-threatening complications and mortality (HR: 1.619 per 10-unit increase in scores, 95% CI: 1.328 to 1.974, *P* < 0.001).

The univariate logistic regression analysis revealed that with each unit increase in the RAI-rev score, the rate of postoperative life-threatening complications and mortality increased by 6.7% (unadjusted OR: 1.067; 95% CI: 1.044 to 1.091; *P* < 0.001). After testing the multicollinearity, 15 variables with *P* < 0.10 that were screened by univariate analyses were included in a multivariable model (see SDC 6 and Table 3). After adjustment for confounding factors, rising RAI-rev scores were independently associated with stepwise increased risk of life-threatening complications and mortality, i.e., every one unit increase in RAI-rev score predicted a 3.0% increase in the odds of this serious adverse outcome (adjusted OR: 1.030; 95% CI: 1.005 to 1.055; *P* = 0.017; Table 3).

**Table 3:** Predictors of postoperative life-threatening complications and mortality

Variables	Univariable analyses		Multivariable analysis ^a^	
	OR (95% CI)	*P* value	OR (95% CI)	*P* value
Body mass index				
18.5–23.9 kg/m^2^	Reference		Reference	
< 18.5 kg/m^2^	3.655 (2.320 to 5.757)	< 0.001	2.938 (1.795 to 4.809)	< 0.001
≥ 24 kg/m^2^	1.296 (0.925 to 1.814)	0.132	1.306 (0.915 to 1.865)	0.142
Revised-Risk Analysis Index scores	1.067 (1.044 to 1.091)	< 0.001	1.030 (1.005 to 1.055)	0.017
ASA classification				
I/II	Reference		Reference	
III	2.559 (1.805 to 3.629)	< 0.001	2.004 (1.389 to 2.893)	< 0.001
IV	9.137 (5.632 to 14.825)	< 0.001	7.202 (4.237 to 12.242)	< 0.001
Hypertension	1.457 (1.067 to 1.988)	0.018	–	–
Coronary heart disease	1.934 (1.370 to 2.729)	< 0.001	–	–
Arrhythmia ^b^	2.109 (1.358 to 3.275)	0.001	–	–
Diabetes mellitus	1.469 (1.055 to 2.045)	0.023	–	–
Chronic pulmonary diseases ^c^	1.506 (0.930 to 2.439)	0.096	–	–
Chronic hepatic dysfunction ^d^	2.483 (1.478 to 4.171)	0.001	–	–
Preoperative infection ^e^	3.897 (2.539 to 5.981)	< 0.001	–	–
Anemia ^f^	2.016 (1.478 to 2.750)	< 0.001	–	–
Hypoalbuminemia ^g^				
None	Reference			
30.0–39.9 g/l	1.961 (1.408 to 2.732)	< 0.001	–	–
< 30.0 g/l	4.787 (2.835 to 8.085)	< 0.001	–	–
Na^+^ < 135.0 mmol/l	2.977 (2.036 to 4.352)	< 0.001	1.942 (1.262 to 2.987)	0.003
Surgery type by Operative Stress Score ^h^				
Low stress	Reference		Reference	
Moderate stress	6.168 (1.497 to 25.420)	0.012	3.324 (0.771 to 14.328)	0.107
High stress	7.503 (1.830 to 30.755)	0.005	4.895 (1.101 to 21.447)	0.037
Very high stress	18.657 (4.078 to 85.355)	< 0.001	8.257 (1.579 to 43.189)	0.012
Duration of surgery (hour)	1.247 (1.141 to 1.363)	< 0.001	1.187 (1.055 to 1.336)	0.004
Emergency surgery	2.870 (1.844 to 4.466)	< 0.001	3.067 (1.769 to 5.316)	< 0.001
Estimated blood loss (100 ml) ^i^	1.046 (1.008 to 1.084)	0.016	–	–
Intra-operative blood transfusion	2.396 (1.503 to 3.821)	< 0.001	–	–

*ASA* American Society of Anesthesiologists, *Na*^*+*^ serum natremia concentration

^a^ Factors with *P* values < 0.10 in univariate analyses or considered clinically important were included in the multivariable logistic regression model. Age, sex, cancer, poor appetite, unintentional weight loss, renal failure, congestive heart failure, shortness of breath, living status, presence of cognitive decline, and functional status were excluded because they were included in the revised-Risk Analysis Index. The multivariable logistic regression analysis was performed with the backward stepwise method. Hosmer-Lemeshow test for goodness of fit of the multivariable model: *χ*^2^ = 12.980, *df* = 8, *P =* 0.113

^b^ Arrhythmia that required medical or interventional therapy

^c^ Include chronic obstructive pulmonary disease and asthma

^d^ Refers to hepatic impairment classified as Child-Pugh class B and C

^e^ Not included in the multivariable logistic regression analysis because of correlation with emergency surgery

^f^ Diagnosed according to the haemoglobin values from the last laboratory test before surgery, male: < 120 g/l, female: < 110 g/l.

^g^ Not included in the multivariable logistic regression analysis because of correlation with poor appetite

^h^ Stratified into five categories of physiologic stress, i.e., very low stress, low stress, moderate stress, high stress, and very high stress [15]. Detailed classification of surgery type by Operative Stress Score is provided in Supplemental Digital Content 4

^i^ Not included in the multivariable logistic regression analysis because of correlation with intra-operative blood transfusion

### Receiver-operating characteristic analysis for MMM

**Fig. 4:**
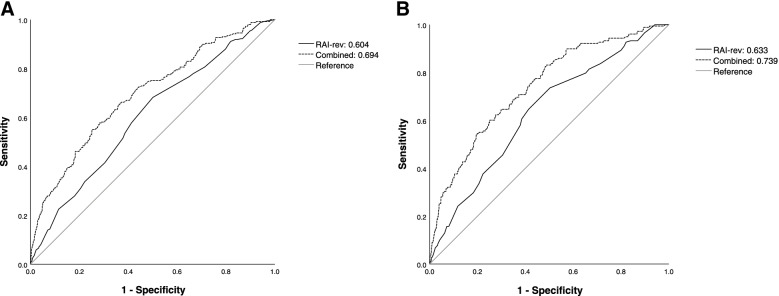
Receiver-operating characteristic curves. **A** The discriminative power of RAI-rev alone (AUC: 0.604; 95% CI: 0.567 to 0.640; *P* < 0.001) and RAI-rev combined with age, gender, ASA classification, operative stress, and urgency status of surgery (AUC: 0.694; 95% CI: 0.659 to 0.729; *P* < 0.001) in predicting the MMM; the combined model had better discrimination than the RAI-rev alone (DeLong’ test: Z = 4.794, *P* < 0.0001). **B** The discriminative power of RAI-rev alone (AUC: 0.633; 95% CI: 0.592 to 0.675; *P* < 0.001) and RAI-rev combined with age, gender, ASA classification, operative stress, and urgency status of surgery (AUC: 0.739; 95% CI: 0.702 to 0.777; *P* < 0.001) in predicting the life-threatening complications and mortality; the combined model had better discrimination than the RAI-rev alone (DeLong’ test: Z = 5.028, *P* < 0.0001)

The AUC of RAI-rev scores in predicting MMM was 0.604 (95% CI: 0.567 to 0.640; *P* < 0.001; Fig. 4A). The sensitivity, specificity, PPV, and NPV for different threshold values of RAI-rev scores were summarised in Table 4. The AUC of the combined model (age, gender, RAI-rev scores, ASA classification, operative stress, and urgency status of surgery) was 0.694 (95% CI: 0.659 to 0.729; *P* < 0.001; Fig. 4A). The combined model had markedly better discrimination than the RAI-rev alone (DeLong’s test: Z = 4.794, *P* < 0.0001).

**Table 4:** Different thresholds of RAI-rev scores

RAI-rev threshold	Frailty prevalence, %	Negative predictive value, %	Positive predictive value, %	Sensitivity, %	Specificity, %
MMM					
30	85.4	93.5	12.5	91.9	15.4
39 ^a^	43.2	91.4	15.5	57.8	58.7
40	40.3	91.0	15.4	53.5	61.4
50	4.7	88.8	20.0	8.1	95.7
60	0.2	88.5	40.0	0.8	99.8
Life-threatening complications and mortality					
30	85.4	96.3	8.7	93.3	15.2
39 ^a^	43.2	95.0	12.0	64.6	58.7
40	40.3	94.7	12.0	60.7	61.5
50	4.7	92.5	17.1	10.1	95.7
60	0.2	92.0	20.0	0.6	99.8

*RAI-rev* Revised-Risk Analysis Index, *MMM* Major morbidity and mortality

^a^ An optimal cutoff value measured by using receiver-operating characteristics curve analysis and Youden’s index

### Receiver-operating characteristic analysis for life-threatening complications and mortality

The AUC of RAI-rev scores in predicting life-threatening complications and mortality was 0.633 (95% CI: 0.592 to 0.675; *P* < 0.001; Fig. 4B). The sensitivity, specificity, PPV, and NPV for different threshold values of RAI-rev scores were detailed in Table 4. The combination of age, gender, RAI-rev scores, ASA classification, operative stress, and urgency status of surgery had improved discriminative power (AUC: 0.739; 95% CI: 0.702 to 0.777; *P* < 0.001; Fig. 4B) than the RAI-rev alone (DeLong’s test: Z = 5.028, *P* < 0.0001).

## Discussion

This retrospective cohort study determined that rising RAI-rev scores were independently associated with stepwise increased risk of MMM in older patients after abdominal surgery. The AUC of the RAI-rev scores was between 0.60 and 0.65 when predicting postoperative MMM or life-threatening complications and death. Compared with the RAI-rev alone, the combination of RAI-rev scores with other baseline risk factors (i.e., age, gender, ASA classification, operative stress, and urgency status of surgery) had significantly improved predictive value for major postoperative complications. Especially for the life-threatening complications and mortality, the combined model showed a moderate predictive value with an AUC of more than 0.70, which is clinically useful in the decision-making process.

It was revealed that postoperative deaths accounted for 7.7% of all deaths worldwide, making it the third leading cause of death [18]. Undoubtedly, major postoperative complications lead to a cascade of perioperative adverse events, including death; furthermore, the occurrence of major complications is associated with poor long-term survival outcomes [19, 20]. The prediction of MMM is the critical first step for clinicians to address the burden of postoperative mortality. In the current study, postoperative MMM occurred in 11.6% of our patients. In previous studies of patients undergoing abdominal surgery, the reported incidence of CD grade III or greater complications ranged from 9.7 to 13.2% [20–22]; the incidence of postoperative MMM in our study population was within this range.

Like the original RAI scoring system, the RAI-rev comprises more comprehensive frailty domains than mFI. The mFI is another well-known deficit accumulation model of frailty and includes merely the domains of comorbidity and functional status [10]. Furthermore, unlike mFI, the RAI-rev is a weighted model with each item having different weights derived from a valid model [9]. When compared with the original RAI, the RAI-rev performed better discrimination and calibration in predicting postoperative mortality [9]. Although the RAI-rev scoring system offered higher weight to male sex, Arya et al. revealed that it also performed robust validity in the female population, confirming its general applicability in clinical settings [9]. Compared with previous studies [8, 9], a larger proportion of patients in our study had high RAI-rev scores. This discrepancy might be attributed to the differences in target patients and clinical settings. To our knowledge, this study is the first to investigate the association of RAI-rev scores with the postoperative MMM, as well as the predictive power of RAI-rev scores for the MMM in older surgical patients.

Our results showed that higher RAI-rev scores were independently associated with an increased risk of major complications, including life-threatening complications and death, in older patients after abdominal surgery. This finding reinforces the available evidence that preoperative frailty is an important predictor of adverse postoperative outcomes [2, 3, 5, 6]. Our results may help perioperative clinicians identify frail patients, predict the postoperative outcomes, and help patients make better informed decisions before surgery. Given the higher risk of major morbidity and mortality in the frail older population, patients and clinicians should adequately evaluate the tradeoff between survival and other potential adverse outcomes (e.g., morbidity, dependent functional status, poor quality of life after surgery) during the preoperative process of shared decision-making. Once frailty is identified, it is essential to determine whether surgical intervention can get the patients to their goals of care. For frail patients, avoiding major morbidity, loss of functional independence, and poor quality of life may sometimes make more sense than longevity. Sensible decision making may, in turn, reduce their mortality. Additionally, our findings can help guide more effective allocation of perioperative care resources and treatment to high-risk patients, thereby improving the safety and quality of surgery among the older population.

Our results demonstrated that the RAI-rev scores lacked good discrimination for the MMM or CD IV or greater complications in older patients undergoing abdominal surgery (AUCs: 0.60–0.65). Previous findings from the studies that had used the frailty tools alone to predict postoperative complications were equally disappointing [14, 23, 24]. This may be attributed to the fact that the etiology of postoperative complications is multifactorial and difficult to predict; the patient-level factors alone could not well explain the variation in complication risk. Thus, additional baseline characteristics or surgical-related factors should also be considered when predicting the risk of postoperative complications. Despite its poor discriminative ability, RAI-rev displayed high NPV at all thresholds; of course, this was also related to the low incidence of postoperative MMM. The high NPV indicated that the RAI-rev possesses a superior ability to exclude patients at low risk of major complications. For patients classified as ‘non-frail’, unnecessary medical modification or intervention (such as planned admission to the ICU after surgery) may be avoided, which can help efficiently allocate perioperative medical resources and reduce hospital costs.

As expected, the combination of RAI-rev with other commonly–used baseline factors (age, gender, ASA classification, operative stress, and urgency status of surgery) showed significantly improved performance to discriminate the risk of major complications, particularly life-threatening complications and mortality (with an AUC above 0.70). A prediction model with AUC exceeding 0.70 may be considered to be useful in clinical decision-making [25]. ASA physical status classification is a traditional preoperative risk stratification tool based on the subjective estimate, reflecting a patient’s physiologic reserve and tolerance to surgical trauma stressors. The operative stress, represented by the OSS, categorizes the surgical procedures based on different degrees of physiologic stress [15]. Given that the OSS system lacks an assessment of the urgency status of surgery, we added the latter into the combined model. Emergency surgery constitutes an important predictor of poor postoperative outcomes due to acute disease processes and inadequate medical optimization before surgery [26]. The above risk factors were all identified as credible predictors for major complications in our multivariable analyses and can be easily acquired in routine clinical practice. Despite its limited ability to predict the MMM risk, the combination of RAI-rev scores with the above risk factors might help clinicians assess the expected risk of life-threatening complications and mortality in older surgical patients. Further studies are required to explore an excellent combined model to predict major postoperative complications.

Besides the retrospective nature, this study had some other limitations. First, our study did not include gynecological patients who underwent abdominal surgery due to the concern about the influence of the special sex distribution of those patients on the RAI-rev score calculation and the final results. However, this might lead to selection bias. Second, the primary endpoint was limited to in-hospital MMM; the occurrence of postdischarge MMM was not observed, which might underestimate the rate of adverse outcomes. Finally, as single-center research, our results may not be extrapolated to patients from other institutions. Despite these, our study for the first time explored the association of RAI-rev scores with postoperative complications and the predictive value of RAI-rev for major complications.

## Conclusion

In conclusion, this study demonstrated that higher RAI-rev scores were associated with an increased risk of postoperative MMM in older patients undergoing abdominal surgery. When combined with age, gender, ASA physical status classification, operative stress, and urgency status of surgery, RAI-rev had better performance in predicting postoperative MMM, particularly the life-threatening complications and mortality. Our findings enable clinicians to better identify high-risk older patients and thus optimize perioperative care and management.

## Supplementary Information

**Additional file 1: Supplemental Digital Content 1.** Revised-Risk Analysis Index Scoring system.

**Additional file 2: Supplemental Digital Content 2.** Clavien**-**Dindo classification of postoperative complications.

**Additional file 3: Supplemental Digital Content 3.** Individual complications and Clavien-Dindo classification.

**Additional file 4: Supplemental Digital Content 4.** Surgical procedures stratified according to Operative Stress Score.

**Additional file 5 Supplemental Digital Content 5.** Factors in association with postoperative MMM (univariate analyses).

**Additional file 6: Supplemental Digital Content 6.** Factors in association with postoperative life-threatening complications and mortality (univariate analyses).

## Data Availability

The data set used and analyzed during the current study is available from the corresponding author on reasonable request.
