# Supplementary material for: The revised-risk analysis index as a predictor of major morbidity and mortality in older patients after abdominal surgery: a retrospective cohort study
Source: BMC Anesthesiol. 2022 Sep 22;22:301. doi: 10.1186/s12871-022-01844-w (PMC9494843; doi:10.1186/s12871-022-01844-w)
Supplement: Supplementary file 1 — Additional file 1: Supplemental Digital Content 1. Revised-Risk Analysis Index Scoring system. [file 12871_2022_1844_MOESM1_ESM.docx]

**Supplemental Digital Content 1** Revised-Risk Analysis Index Scoring system [9]

| Variable | RAI-rev | |
| --- | --- | --- |
| Male sex | 3 | |
| Weight loss | 4 | |
| Poor appetite | 4 | |
| Renal failure | 8 | |
| Congestive heart failure | 5 | |
| Shortness of breath at rest | 3 | |
| Residence other than independent living | 1 | |
| Age* cancer | Without cancer | With cancer |
| Age |  |  |
| 65–69 | 20 | 34 |
| 70–74 | 22 | 34 |
| 75–79 | 24 | 35 |
| 80–84 | 26 | 35 |
| 85–89 | 28 | 36 |
| 90–94 | 30 | 36 |
| 95–99 | 32 | 37 |
| ADL* cognitive decline | Without cognitive decline | With cognitive decline |
| Totally dependent | 14 | 16 |
| Partially dependent | 7 | 11 |
| Independent | 0 | 5 |

Total score (range): 0 to 81
