# Supplementary material for: The revised-risk analysis index as a predictor of major morbidity and mortality in older patients after abdominal surgery: a retrospective cohort study
Source: BMC Anesthesiol. 2022 Sep 22;22:301. doi: 10.1186/s12871-022-01844-w (PMC9494843; doi:10.1186/s12871-022-01844-w)
Supplement: Supplementary file 2 — Additional file 2: Supplemental Digital Content 2. Clavien-Dindo classification of postoperative complications. [file 12871_2022_1844_MOESM2_ESM.docx]

**Supplemental Digital Content 2** Clavien**-**Dindo classification of postoperative complications [16]

| Grade | Definition |
| --- | --- |
| Grade I | Any deviation from the normal postoperative course without the need for special interventions; complications that are managed with routine medication (such as analgesics, antiemetics, antipyretics, diuretics, and electrolytes) or physiotherapy, and wound infections that are managed at the bedside are classified as Grade I. |
| Grade II | Requiring total parenteral nutrition, blood transfusions, or pharmacological treatment with drugs other than those allowed for Grade I complications. |
| Grade III | Requiring surgical, endoscopic, or radiological intervention. |
| Grade IV | Life-threatening complications requiring intermediate care (IC)/intensive care unit (ICU) management excluding transient ischemic attacks. |
| Grade V | Death of a patient. |
