# Supplementary material for: The revised-risk analysis index as a predictor of major morbidity and mortality in older patients after abdominal surgery: a retrospective cohort study
Source: BMC Anesthesiol. 2022 Sep 22;22:301. doi: 10.1186/s12871-022-01844-w (PMC9494843; doi:10.1186/s12871-022-01844-w)
Supplement: Supplementary file 3 — Additional file 3: Supplemental Digital Content 3. Individual complications and Clavien-Dindo classification. [file 12871_2022_1844_MOESM3_ESM.docx]

**Supplemental Digital Content 3** Individual complications and Clavien-Dindo classification

| Complications | All patients (*n*=2225) |
| --- | --- |
| Cardiovascular complications, *n* (%) | 107 (4.8%) |
| Acute coronary syndrome ^a^ | 28 (1.3%) |
| New-onset arrhythmia ^b^ | 26 (1.2%) |
| Circulatory insufficiency ^c^ | 57 (2.6%) |
| Acute heart failure or exacerbation of congestive heart failure ^d^ | 38 (1.7%) |
| Cardiac arrest ^e^ | 4 (0.2%) |
| Respiratory complications, *n* (%) | 91 (4.1%) |
| Pulmonary infection ^f^ | 29 (1.3%) |
| Respiratory failure ^g^ | 69 (3.1%) |
| Atelectasis ^h^ | 6 (0.3%) |
| Pleural effusion ^i^ | 9 (0.4%) |
| Bronchospasm ^j^/asthma attack ^k^ | 7 (0.3%) |
| Neurological complications, *n* (%) | 30 (1.3%) |
| Stroke ^l^ | 11 (0.5%) |
| Delirium ^m^ | 14 (0.6%) |
| Pulmonary encephalopathy ^n^ | 5 (0.2%) |
| Renal complications, *n* (%) | 18 (0.8%) |
| Acute renal failure ^o^ | 18 (0.8%) |
| Thrombotic complications, *n* (%) | 12 (0.5%) |
| Pulmonary embolism ^p^ | 5 (0.2%) |
| Disseminated intravascular coagulation ^q^ | 7 (0.3%) |
| Surgery-related complications, *n* (%) | 115 (5.2%) |
| Intra-abdominal/pelvic abscess ^r^ | 39 (1.8%) |
| Wound dehiscence ^s^ | 3 (0.1%) |
| Anastomotic leak ^t^ | 31 (1.4%) |
| Anastomotic stenosis ^u^ | 4 (0.2%) |
| Ileus ^v^ | 48 (2.2%) |
| Surgical haemorrhage ^w^ | 23 (1.0%) |
| Rejection of hernia mesh ^x^ | 1(0.04%) |
| Infectious complications, *n* (%) | 46 (2.1%) |
| Sepsis ^y^ | 46 (2.1%) |
| Complications according to Clavien-Dindo classification |  |
| Grade III | 80 (3.6%) |
| Grade IV | 152 (6.8%) |
| Grade V | 26 (1.2%) |

Data are *n* (%).

^a^ Include acute myocardial infarction and unstable angina, which were confirmed by clinical symptoms, electrocardiographic changes, imaging evidence, serum cardiac troponin I concentration, and requirement of IC/ICU management.

^b^ Confirmed by 12-lead electrocardiogram and necessitated cardioversion or IC/ICU management.

^c^ Requirement of inotropics or vasopressors for more than 24 hours after surgery and necessitated IC/ICU management.

^d^ Diagnosed by the presence of new-onset orthopnea with evidence of fluid retention (i.e., elevated jugular venous pressure, evidence of pulmonary oedema, and/or peripheral oedema), an elevated plasma brain natriuretic peptide of above 400 pg ml^-1^, and necessitated IC/ICU management.

^e^ The absence of large artery pulsation and heart sound, subsequent loss of consciousness, respiratory arrest, dilated pupils and even death, caused by the sudden stop of heart beating.

^f^ Pneumonia that required bronchoscopic aspiration or mechanical ventilation, or pneumonia that caused sepsis or multiple organ failure.

^g^ *P*aO_2_ <60 mmHg on room air, a ratio of *P*aO_2_ to inspired oxygen fraction <300, or arterial oxyhemoglobin saturation measured with pulse oximetry <90% and requiring oxygen therapy and IC/ICU management, or mechanical ventilation.

^h^ Confirmed by lung opacification with a shift of the mediastinum, hilum, or hemidiaphragm toward the affected area, compensatory overinflation in the adjacent non-atelectatic lung, and requirement of mechanical ventilation or thoracentesis including drain placement.

^i^ Confirmed by chest X-ray demonstrating blunting of the costophrenic angle, loss of the sharp silhouette of the ipsilateral hemidiaphragm in the upright position, a hazy opacity in one hemithorax with preserved vascular shadows in the supine position, evidence of displacement of adjacent anatomical structures, and requirement of mechanical ventilation or thoracentesis including drain placement.

^j^ Confirmed by new-onset expiratory wheezing, necessitated treatment with mechanical ventilation, or bronchodilators and IC/ICU management.

^k^ Diagnosed according to clinical signs, necessitated mechanical ventilation, or inhaled bronchodilator therapy and necessitated IC/ICU management.

^l^ Persisted new focal neurologic deficit confirmed by neurologic imaging, requiring radiological/surgical intervention or IC/ICU management.

^m^ Diagnosed by psychiatric symptoms of inattention and thinking disorder, or inattention and altered level of consciousness, with an acute onset or a fluctuation course, and necessitated IC/ICU management.

^n^ Neuropsychiatric syndrome caused by carbon dioxide accumulation and hypoxia due to respiratory failure and ruled out other causes of neuropsychiatric disorders, requiring IC/ICU management.

^o^ New onset renal failure that required renal replacement therapy.

^p^ Hypotension or shock suspected of pulmonary embolism requiring IC/ICU level management, and meeting one of the following: filling defect in any branch of the pulmonary artery in computed tomographic pulmonary angiogram or right ventricular overload in the echocardiogram. For suspected pulmonary embolism without symptoms, positive finding in computed tomographic pulmonary angiogram was required for diagnosis.

^q^ Diagnosed by abnormal bleeding symptoms, and more than three anomalies in the following items: platelet <100×10^9^ l^-1^ or progressive decline; fibrinogen <1.5 g l^-1^ or progressive decline or >4 g l^-1^; plasma fibrin degradation product (FDP) >20 mg l^-1^ or D-dimer level increased or positive, or 3P test (plasma protamine paracoagulation test) positive; prothrombin time (PT) shorter or longer than 3 s or activated partial thromboplastin time (APTT) shorter or longer than 10 s.

^r^ Confirmed by imaging examination, or the purulent fluid extracted by puncture and bacteria cultured from the fluid and necessitated image-guided drain placement/paracentesis/secondary surgery; or intra-abdominal/pelvic abscess that caused sepsis or at least one organ failure.

^s^ Wound rupture that required secondary suturing or antibiotics.

^t^ Diagnosed by extravasation of contrast agent in the body cavity or retroperitoneal space during imaging examination, or perianastomotic abscess, and necessitated image-guided drain placement/paracentesis /secondary surgery; or anastomotic leak that caused sepsis or at least one organ failure.

^u^ Diagnosis supported by imaging examination or endoscopy and ruled out the presence of gastrointestinal dysfunction and mechanical obstruction, requiring balloon dilation, stenting or secondary surgery.

^v^ Diagnosed by lack of bowel movement, flatulence, and requirement of nasoenteric tube placement or secondary surgery; or extensive intestinal necrosis, with at least one organ failure or sepsis.

^w^ Bleeding after surgery that required secondary surgical hemostasis.

^x^ Diagnosed by the symptoms of non-healing wounds, exudation, abdominal pain, and fever (ruled out the presence of incision infection or subcutaneous effusion), and necessitated surgical intervention.

^y^ Two or more criteria of systemic inflammatory response syndrome, with known infection and new-onset dysfunction of at least one organ/system.
