# Supplementary material for: The revised-risk analysis index as a predictor of major morbidity and mortality in older patients after abdominal surgery: a retrospective cohort study
Source: BMC Anesthesiol. 2022 Sep 22;22:301. doi: 10.1186/s12871-022-01844-w (PMC9494843; doi:10.1186/s12871-022-01844-w)
Supplement: Supplementary file 4 — Additional file 4: Supplemental Digital Content 4. Surgical procedures stratified according to Operative Stress Score. [file 12871_2022_1844_MOESM4_ESM.docx]

**Supplemental Digital Content 4** Surgical procedures stratified according to Operative Stress Score ^15^

| Category, Procedure Type | All patients (*n*=2225) |
| --- | --- |
| **Category 1, Very Low Stress** |  |
| All | 0 (0.0%) |
| **Category 2, Low Stress** |  |
| All | 157 (7.1%) |
| Appendectomy, unruptured, laparoscopic or open | 53 (2.4%) |
| Inguinal hernia, laparoscopic or open | 43 (1.9%) |
| Incisional hernia repair, reducible, laparoscopic or open | 6 (0.3%) |
| Umbilical hernia repair, reducible | 4 (0.2%) |
| Partial nephrectomy, laparoscopic | 51(2.3%) |
| **Category 3, Moderate Stress** |  |
| All | 936 (42.1%) |
| Cholecystectomy, laparoscopic or open with or without intraoperative cholangiogram | 67 (3.0%) |
| Hepatic cyst fenestration, laparoscopic | 6 (0.3%) |
| Gastric restrictive procedure, longitudinal gastrectomy, laparoscopic | 6 (0.3%) |
| Gastrostomy, laparoscopic | 8 (0.4%) |
| Closure of enterostomy, large or small intestine | 36 (1.6%) |
| Colectomy, laparoscopic | 202 (9.1%) |
| Enterectomy, enteroenterostomy | 74 (3.3%) |
| Enterolysis | 10 (0.4%) |
| Suture of gastrointestinal tract for perforation | 8 (0.4%) |
| Splenectomy | 4 (0.2%) |
| Radical nephrectomy, open or laparoscopic | 159 (7.1%) |
| Nephrectomy with total ureterectomy, laparoscopic | 138 (6.2%) |
| Prostatectomy, laparoscopic, suprapubic, 1 or 2 stages | 162 (7.3%) |
| Adrenalectomy( except pheochromocytoma) | 56 (2.5%) |
| **Category 4, High Stress** |  |
| All | 1065 (47.9%) |
| Colectomy, open | 105 (4.7%) |
| Gastrectomy | 201 (9.0%) |
| Hepatectomy, resection of liver; partial lobectomy | 53 (2.4%) |
| Proctectomy, laparoscopic or open | 304 (13.7%) |
| Pancreatectomy, distal subtotal, with or without splenectomy; without pancreatojejunostomy | 28 (1.3%) |
| Excision of huge primary retroperitoneal tumor | 22 (1.0%) |
| Nephrectomy, partial | 38 (1.7%) |
| Nephrectomy, radical, with vena caval thrombectomy | 8 (0.4%) |
| Pheochromocytoma | 11 (0.5%) |
| Complete cystectomy, with continent diversion, including using a  segment of ileum to construct neobladder, or cutaneous ureterostomy | 275 (12.4%) |
| Prostatectomy, retropubic radical, with bilateral pelvic lymphadenectomy | 20 (0.9%) |
| **Category 5, Very High Stress** |  |
| All | 67 (3.0%) |
| Pancreaticoduodenectomy (Whipple-type procedure) | 67 (3.0%) |

Data are *n* (%).
