# Supplementary material for: The revised-risk analysis index as a predictor of major morbidity and mortality in older patients after abdominal surgery: a retrospective cohort study
Source: BMC Anesthesiol. 2022 Sep 22;22:301. doi: 10.1186/s12871-022-01844-w (PMC9494843; doi:10.1186/s12871-022-01844-w)
Supplement: Supplementary file 5 — Additional file 5 Supplemental Digital Content 5. Factors in association with postoperative MMM (univariate analyses). [file 12871_2022_1844_MOESM5_ESM.docx]

**Supplemental Digital Content 5** Factors in association with postoperative MMM (univariate analyses)

| Variables ^a^ | Number | OR (95% CI) | *P* value |
| --- | --- | --- | --- |
| Body mass index |  |  |  |
| 18.5-23.9 kg m^-2^ | 1141 | Reference |  |
| <18.5 kg m^-2^ | 162 | 2.931 (1.938 to 4.434) | **<0.001** |
| ≥24 kg m^-2^ | 922 | 1.323 (1.000 to 1.750) | 0.050 |
| RAI-rev scores | 2225 | 1.053 (1.034 to 1.072) | **<0.001** |
| ASA classification |  |  |  |
| I/II | 1234 | Reference |  |
| III | 890 | 1.905 (1.435 to 2.528) | **<0.001** |
| IV | 101 | 6.464 (4.191 to 9.971) | **<0.001** |
| Current smoker/quit ≤ 7 days | 276 | 1.300 (0.901 to 1.876) | 0.161 |
| Current alcoholism | 101 | 1.133 (0.623 to 2.059) | 0.682 |
| Hypertension | 1122 | 1.169 (0.901 to 1.517) | 0.239 |
| Coronary heart disease | 403 | 1.584 (1.167 to 2.151) | **0.003** |
| Arrhythmia ^b^ | 187 | 1.800 (1.210 to 2.676) | **0.004** |
| Peripheral vascular disease | 236 | 1.433 (0.978 to 2.098) | **0.065** |
| Diabetes mellitus | 554 | 1.386 (1.043 to 1.842) | **0.024** |
| Chronic pulmonary diseases ^c^ | 188 | 1.187 (0.763 to 1.849) | 0.447 |
| Obstructive sleep apnea ^d^ | 85 | 1.673 (0.943 to 2.968) | **0.079** |
| Previous stroke | 375 | 1.249 (0.899 to 1.736) | 0.184 |
| Stroke with deficits ^e^ | 92 | 1.779 (1.034 to 3.064) | **0.038** |
| Mental disorders ^f^ | 48 | 1.310 (0.581 to 2.952) | 0.515 |
| Visual/hearing impairment | 86 | 1.377 (0.752 to 2.521) | 0.300 |
| Chronic hepatic dysfunction ^g^ | 113 | 2.164 (1.352 to 3.466) | **0.001** |
| Connective tissue disease | 37 | 0.632 (0.149 to 2.692) | 0.535 |
| Chronic corticosteroid therapy ^h^ | 77 | 1.578 (0.856 to 2.906) | 0.143 |
| Hyper-/hypothyroidism | 43 | 1.766 (0.810 to 3.851) | 0.152 |
| Preoperative infection | 141 | 2.999 (2.010 to 4.475) | **<0.001** |
| Anemia ^i^ | 670 | 1.703 (1.304 to 2.224) | **<0.001** |
| Blood coagulation disorder | 44 | 1.209 (0.506 to 2.888) | 0.670 |
| History of DVT or PE | 15 | 1.174 (0.263 to 5.233) | 0.833 |
| Dyslipidemia | 1136 | 1.220 (0.940 to 1.583) | 0.136 |
| Hypoalbuminemia |  |  |  |
| None | 1215 | Reference |  |
| 30.0–39.9 g l^-1^ | 902 | 1.541 (1.172 to 2.025) | **0.002** |
| <30.0 g l^-1^ | 108 | 3.154 (1.947 to 5.109) | **<0.001** |
| Na^+^ <135.0 mmol l^-1^ | 228 | 1.992 (1.393 to 2.851) | **<0.001** |
| Surgery type by Operative Stress Score ^j^ |  |  |  |
| Low stress | 157 | Reference |  |
| Moderate stress | 936 | 4.119 (1.491 to 11.378) | **0.006** |
| High stress | 1065 | 5.980 (2.182 to 16.389) | **0.001** |
| Very high stress | 67 | 15.141 (4.911 to 46.679) | **<0.001** |
| Duration of surgery (hour) | 2225 | 1.214 (1.123 to 1.312) | **<0.001** |
| Type of anaesthesia |  |  |  |
| General | 1225 | Reference |  |
| Regional/combined regional-general | 1000 | 0.928 (0.716 to 1.205) | 0.576 |
| Emergency surgery | 153 | 2.160 (1.429 to 3.266) | **<0.001** |
| Estimated blood loss (100 ml) | 2225 | 1.052 (1.019 to1.086) | **0.002** |
| Intra-operative blood transfusion | 149 | 2.444 (1.627 to 3.672) | **<0.001** |

*P* values in bold indicate <0.10.

^a^ The 11 baseline variables included in the RAI-rev were not separately enrolled in univariable analyses.

^b^ Arrhythmia that required medical or interventional therapy.

^c^ Include chronic obstructive pulmonary disease and asthma.

^d^ Diagnosed by previous polysomnography, or history inquiry and physical examination, and/or STOP-Bang/Berlin questionnaire.

^e^ Excludes vascular dementia.

^f^ Include diagnosed depression, anxiety, schizophrenia, phobia, and hallucination.

^g^ Refers to hepatic impairment classified as Child-Pugh class B and C.

^h^ With a duration of >1 month.

^i^ Diagnosed according to the haemoglobin values from the last laboratory test before surgery, male: <120 g l^-1^, female: <110 g l^-1^.

^j^ Stratified into five categories of physiologic stress, i.e., very low stress, low stress, moderate stress, high stress, and very high stress.^15^ Detailed classification of surgery type by Operative Stress Score is provided in Supplemental Digital Content 4.

RAI-rev, revised Risk Analysis Index; ASA, American Society of Anesthesiologists; DVT, deep venous thrombosis; PE, pulmonary embolism; Na^+^, serum natremia concentration.
