# Supplementary material for: The revised-risk analysis index as a predictor of major morbidity and mortality in older patients after abdominal surgery: a retrospective cohort study
Source: BMC Anesthesiol. 2022 Sep 22;22:301. doi: 10.1186/s12871-022-01844-w (PMC9494843; doi:10.1186/s12871-022-01844-w)
Supplement: Supplementary file 6 — Additional file 6: Supplemental Digital Content 6. Factors in association with postoperative life-threatening complications and mortality (univariate analyses). [file 12871_2022_1844_MOESM6_ESM.docx]

**Supplemental Digital Content 6** Factors in association with postoperative life-threatening complications and mortality (univariate analyses)

| Variables ^a^ | Number | OR (95% CI) | *P* value |
| --- | --- | --- | --- |
| Body mass index |  |  |  |
| 18.5-23.9 kg m^-2^ | 1141 | Reference |  |
| <18.5 kg m^-2^ | 162 | 3.655 (2.320 to 5.757) | **<0.001** |
| ≥24 kg m^-2^ | 922 | 1.296 (0.925 to 1.814) | 0.132 |
| RAI-rev scores | 2225 | 1.067 (1.044 to 1.091) | **<0.001** |
| ASA classification |  |  |  |
| I/II | 1234 | Reference |  |
| III | 890 | 2.559 (1.805 to 3.629) | **<0.001** |
| IV | 101 | 9.137 (5.632 to 14.825) | **<0.001** |
| Current smoker/quit ≤ 7 days | 276 | 1.354 (0.885 to 2.071) | 0.162 |
| Current alcoholism | 101 | 1.432 (0.751 to 2.732) | 0.275 |
| Hypertension | 1122 | 1.457 (1.067 to 1.988) | **0.018** |
| Coronary heart disease | 403 | 1.934 (1.370 to 2.729) | **<0.001** |
| Arrhythmia ^b^ | 187 | 2.109 (1.358 to 3.275) | **0.001** |
| Peripheral vascular disease | 236 | 1.422 (0.910 to 2.221) | 0.122 |
| Diabetes mellitus | 554 | 1.469 (1.055 to 2.045) | **0.023** |
| Chronic pulmonary diseases ^c^ | 188 | 1.506 (0.930 to 2.439) | **0.096** |
| Obstructive sleep apnea ^d^ | 85 | 1.381 (0.680 to 2.805) | 0.372 |
| Previous stroke | 375 | 1.181 (0.799 to 1.747) | 0.404 |
| Stroke with deficits ^e^ | 92 | 1.426 (0.726 to 2.802) | 0.303 |
| Mental disorders ^f^ | 48 | 1.347 (0.527 to 3.445) | 0.534 |
| Visual/hearing impairment | 86 | 1.188 (0.564 to 2.501) | 0.650 |
| Chronic hepatic dysfunction ^g^ | 113 | 2.483 (1.478 to 4.171) | **0.001** |
| Connective tissue disease | 37 | 0.653 (0.156 to 2.739) | 0.560 |
| Chronic corticosteroid therapy ^h^ | 77 | 1.550 (0.760 to 3.161) | 0.228 |
| Hyper-/hypothyroidism | 43 | 1.528 (0.594 to 3.932) | 0.379 |
| Preoperative infection | 141 | 3.897 (2.539 to 5.981) | **<0.001** |
| Anemia ^i^ | 670 | 2.016 (1.478 to 2.750) | **<0.001** |
| Blood coagulation disorder | 44 | 1.488 (0.579 to 3.824) | 0.409 |
| History of DVT or PE | 15 | 0.820 (0.107 to 6.275) | 0.849 |
| Dyslipidemia | 1136 | 1.106 (0.814 to 1.504) | 0.520 |
| Hypoalbuminemia |  |  |  |
| None | 1215 | Reference |  |
| 30.0–39.9 g l^-1^ | 902 | 1.961 (1.408 to 2.732) | **<0.001** |
| <30.0 g l^-1^ | 108 | 4.787 (2.835 to 8.085) | **<0.001** |
| Na^+^ <135.0 mmol l^-1^ | 228 | 2.977 (2.036 to 4.352) | **<0.001** |
| Surgery type by Operative Stress Score ^j^ |  |  |  |
| Low stress | 157 | Reference |  |
| Moderate stress | 936 | 6.168 (1.497 to 25.420) | **0.012** |
| High stress | 1065 | 7.503 (1.830 to 30.755) | **0.005** |
| Very high stress | 67 | 18.657 (4.078 to 85.355) | **<0.001** |
| Duration of surgery (hour) | 2225 | 1.247 (1.141 to 1.363) | **<0.001** |
| Type of anaesthesia |  |  |  |
| General | 1225 | Reference |  |
| Regional/combined regional-general | 1000 | 0.922 (0.678 to 1.254) | 0.603 |
| Emergency surgery | 153 | 2.870 (1.844 to 4.466) | **<0.001** |
| Estimated blood loss (100 ml) | 2225 | 1.046 (1.008 to 1.084) | **0.016** |
| Intra-operative blood transfusion | 149 | 2.396 (1.503 to 3.821) | **<0.001** |

*P* values in bold indicate <0.10.

^a^ The 11 baseline variables included in the RAI-rev were not separately enrolled in univariable analyses.

^b^ Arrhythmia that required medical or interventional therapy.

^c^ Include chronic obstructive pulmonary disease and asthma.

^d^ Diagnosed by previous polysomnography, or history inquiry and physical examination, and/or STOP-Bang/Berlin questionnaire.

^e^ Excludes vascular dementia.

^f^ Include diagnosed depression, anxiety, schizophrenia, phobia, and hallucination.

^g^ Refers to hepatic impairment classified as Child-Pugh class B and C.

^h^ With a duration of >1 month.

^i^ Diagnosed according to the haemoglobin values from the last laboratory test before surgery, male: <120 g l^-1^, female: <110 g l^-1^.

^j^ Stratified into five categories of physiologic stress, i.e., very low stress, low stress, moderate stress, high stress, and very high stress.^15^ Detailed classification of surgery type by Operative Stress Score is provided in Supplemental Digital Content 4.

RAI-rev, revised Risk Analysis Index; ASA, American Society of Anesthesiologists; DVT, deep venous thrombosis; PE, pulmonary embolism; Na^+^, serum natremia concentration.
